# The Lifelines Cohort Study: Prevalence of Tinnitus Associated Suffering and Behavioral Outcomes in Children and Adolescents

**DOI:** 10.1097/AUD.0000000000001538

**Published:** 2024-07-10

**Authors:** Sebastiaan M. Meijers, Jessica H. J. de Ruijter, Robert J. Stokroos, Adriana L. Smit, Inge Stegeman

**Affiliations:** 1 Department of Otorhinolaryngology and Head & Neck Surgery, University Medical Center Utrecht, Utrecht, The Netherlands; 2 Brain Center, University Medical Center Utrecht, Utrecht, The Netherlands.

**Keywords:** Adolescents, Behavior, Children, Externalizing problems, Impact on daily life, Internalizing problems, Tinnitus

## Abstract

**Objectives:**

Tinnitus in children and adolescents is relatively unexplored territory. The available literature is limited and the reported prevalence of tinnitus suffering varies widely due to the absence of a definition for pediatric tinnitus. The impact on daily life seems to be lower than in the adult population. It is unclear if children who suffer from tinnitus, like adults, also experience psychological distress like anxiety or depressive symptoms. A better understanding of tinnitus in children and its impact on daily life could provide more insight into the actual size of the problem and could give direction for future studies to investigate the cause of progression of tinnitus.

**Design:**

A cross-sectional study was performed using the Dutch Lifelines population-based cohort of people living in the north of the Netherlands. A total of 4964 children (4 to 12 years of age) and 2506 adolescents (13 to 17 years of age) were included. The presence of tinnitus suffering and behavioral outcomes were assessed with a single-item question and the Child Behavioral Checklist or the Youth Self Report questionnaire respectively. The associations of behavioral outcomes and tinnitus suffering were analyzed using univariate binary regressions.

**Results:**

The prevalence of tinnitus suffering in children was 3.3 and 12.8% in adolescents. Additionally, 0.3% of the children and 1.9% of the adolescents suffered a lot or extremely of their tinnitus. Externalizing and internalizing problems were associated with tinnitus in adolescents. Internalizing problems were associated with tinnitus in children.

**Conclusions:**

The prevalence of tinnitus suffering in this sample of the general population is comparable to other population-based studies. A low percentage of children (0.3%) or adolescents (1.9%) suffered a lot or extremely of their tinnitus. Tinnitus suffering is associated with all behavioral outcome subscales in adolescents and with internalizing problems in children, although the effect sizes were very small. Future research should focus on achieving a consensus for the definition of pediatric tinnitus and on the development of a validated outcome measure.

## INTRODUCTION

Tinnitus is a common phenomenon in adults with a prevalence of 5.1 to 42.7% in Western societies (McCormack et al. 2016; Oosterloo et al. 2021; Rademaker et al. 2021). Only a part of those with tinnitus experience tinnitus-related emotional distress, cognitive dysfunction and/or autonomic arousal and/or behavioral changes, and functional disability which is defined as having a tinnitus disorder (De Ridder et al. 2021).

Unlike research on tinnitus in adults, literature regarding the prevalence of tinnitus and impact on daily life in children and adolescents is rather limited. Rosing et al. (2016) published a systematic review in 2016 in which they reported a prevalence of tinnitus of 4.7 to 46% in children with normal hearing and a prevalence of 23.5 to 62.6% in children with hearing loss. The prevalence of more severe tinnitus was reported to range from 0.6 to 49.2% of those with tinnitus using different definitions. Other more recent population-based tinnitus studies in children reported a prevalence of severe tinnitus between 3.1 and 66.9% (Båsjö et al. 2016; Humphriss et al. 2016; Kim 2018; Nemholt et al. 2020; Rhee et al. 2020; Raj-Koziak et al. 2021). The wide ranges reported could be explained by differences in study design, study population, the used definition of tinnitus, and the used question or questionnaire to assess tinnitus and its impact (Rosing et al. 2016; Smith et al. 2019). (See supplementary file: overview of published studies in English literature since Rosing et al. in Supplemental Digital Content, http://links.lww.com/EANDH/B434, and for the used descriptions of tinnitus and “severe” tinnitus.)

Unlike adults, for which there seems a general consensus on the definition of tinnitus and having a tinnitus disorder, this is not the case in for the pediatric population.

The lack of instruments to measure the prevalence and impact of tinnitus on daily life specifically for children complicates tinnitus research in this patient category and the need for such a questionnaire was also highlighted in the 2020 UK National Institute for Health and Care Excellence guideline on tinnitus assessment and management (National Institute of Health and Care Excellence [NICE] 2020). Obtaining reliable answers from young children is challenging because of the risk of misunderstood questions, difficulties for young children to estimate time, to recall events, and the absence of a pediatric tinnitus questionnaire (Baguley & McFerran 1999; Rosing et al. 2016; Smith et al. 2019; Nemholt et al. 2020). Besides this, children rarely spontaneously report their tinnitus and it is unknown what percentage of the children with tinnitus are so bothered by it that they may need professional medical help to cope with it (Savastano 2007; Baguley et al. 2013; Nemholt et al. 2020).

Nevertheless, the issues mentioned about assessing prevalence, there is not much known about the relation between having tinnitus in children and their behavior. Is the presence of tinnitus related to having feelings of anxiety, depression, stress, or sleeping problems as seen in the adult population (Trevis et al. 2018; Salazar et al. 2019; Meijers et al. 2022)? So far, investigation of the behavioral domain is scarce.

To date, several observational studies reported elevated anxiety levels (Kentish et al. 2000; Brunnberg et al. 2008; Kim et al. 2012; Adegbiji et al. 2018; Chan et al. 2018) and depressive symptoms (Adegbiji et al. 2018; Kim 2018; Rhee et al. 2020) in children with tinnitus but with nonvalidated methods. Stress, attention problems, sleeping problems, and somatic complaints have also been reported (Brunnberg et al. 2008; Kim et al. 2012; Adegbiji et al. 2018; Chan et al. 2018; Rhee et al. 2020). Only Holgers and Juul (2006) and Kim et al. (2012) used validated questionnaires to investigate behavior. Holgers and Juul used the Hospital Anxiety and Depression Scale depression and Hospital Anxiety and Depression Scale anxiety questionnaire and found that both had a moderate correlation with tinnitus which was measured with the Tinnitus Severity Questionnaire (Zigmond & Snaith 1983; Coles et al. n.d.). Kim et al. used the State and Trait Anxiety Inventory for Children and found that children who more frequently report the presence of tinnitus also had significantly higher level of trait anxiety in comparison to the children without tinnitus.

A major drawback in these studies is the lack of a frequently used, validated questionnaire to measure behavioral outcomes. Two of the most frequently used questionnaires; the Child behavioral Checklist (CBCL) and Youth Self Report (YSR) have to date not been used to study pediatric tinnitus patients (Achenbach 1999). CBCL and YSR questionnaires are used worldwide and are validated in many languages. The use of such a questionnaire and/or additional Patient-Reported Outcome Measurement Information System (PROMIS) questionnaires would ease reproducibility, comparability and could aid in the development of a future pediatric tinnitus questionnaire.

Therefore, we aim to: (1) evaluate the prevalence and severity of tinnitus suffering in children and adolescents in the general Dutch population, and (2) investigate the associations between behavioral outcomes (e.g., anxiety, depressive symptoms, fatigue, social behavior) and tinnitus severity in children and adolescents. Tinnitus in this study is defined as tinnitus with any degree of suffering from ringing in the ears. Better understanding of tinnitus in children and the impact it has on daily life could provide more insight into the actual size of the problem and could give direction for future studies to investigate the relation or progression from tinnitus to tinnitus disorder.

## MATERIALS AND METHODS

### Study Design

Participants of the Dutch Lifelines Cohort Study under the age of 18 were studied. Lifelines is a multidisciplinary prospective population-based cohort study examining in a unique three-generation design the health and health-related behaviors of 167,729 persons living in the North of the Netherlands. It uses a broad range of investigative procedures in assessing the biomedical, socio-demographic, behavioral, physical, and psychological factors that contribute to the health and disease of the general population, with a special focus on multimorbidity and complex genetics. All inhabitants of the Netherlands are registered with a general practitioner (GP) and all GPs of the three northern provinces of the Netherlands were invited to participate in the LifeLines Cohort study. Of those invited, 73% (n = 562/812) of the GPs agreed to participate in this study, and in total 333,307 participants aged 25 to 50 years were invited by their GP. Participants were stimulated to invite family members to join the study. It was also possible for participants to self-register. All children were recruited via their parents. These invitations took place between 2006 and 2013. In total 167,729 participants agreed to participate, of which 8147 were under the age of 18 years at assessment “2A.” For the adult population, the LifeLines study population is broadly representative for the northern part of the Netherlands (Klijs et al. 2015). For the pediatric population, no data is available. Exclusion criteria for Lifelines participation were a severe mental illness, a short life expectancy (<5 years) at time of inclusion, insufficient knowledge of the Dutch language to complete the questionnaires, and being not able to visit their GP (Scholtens et al. 2015). All questionnaires were in Dutch. The Lifelines Cohort Study is approved by the Medical Ethical Committee of the University Medical Center Groningen, The Netherlands under number 2007/152 and is performed according to the principles of the Declaration of Helsinki. The methods and a description of the used measurements within the Lifelines Study are extensively described (Scholtens et al. 2015; Sijtsma et al. 2022). For this study, the Strengthening the Reporting of Observational Studies in Epidemiology (STROBE) statement guideline for reporting of observational studies was used. (Appendix 1: STROBE checklist in Supplemental Digital Content, http://links.lww.com/EANDH/B435 [von Elm et al. 2007].)

### Participants and Data Collection

**Fig. 1.**
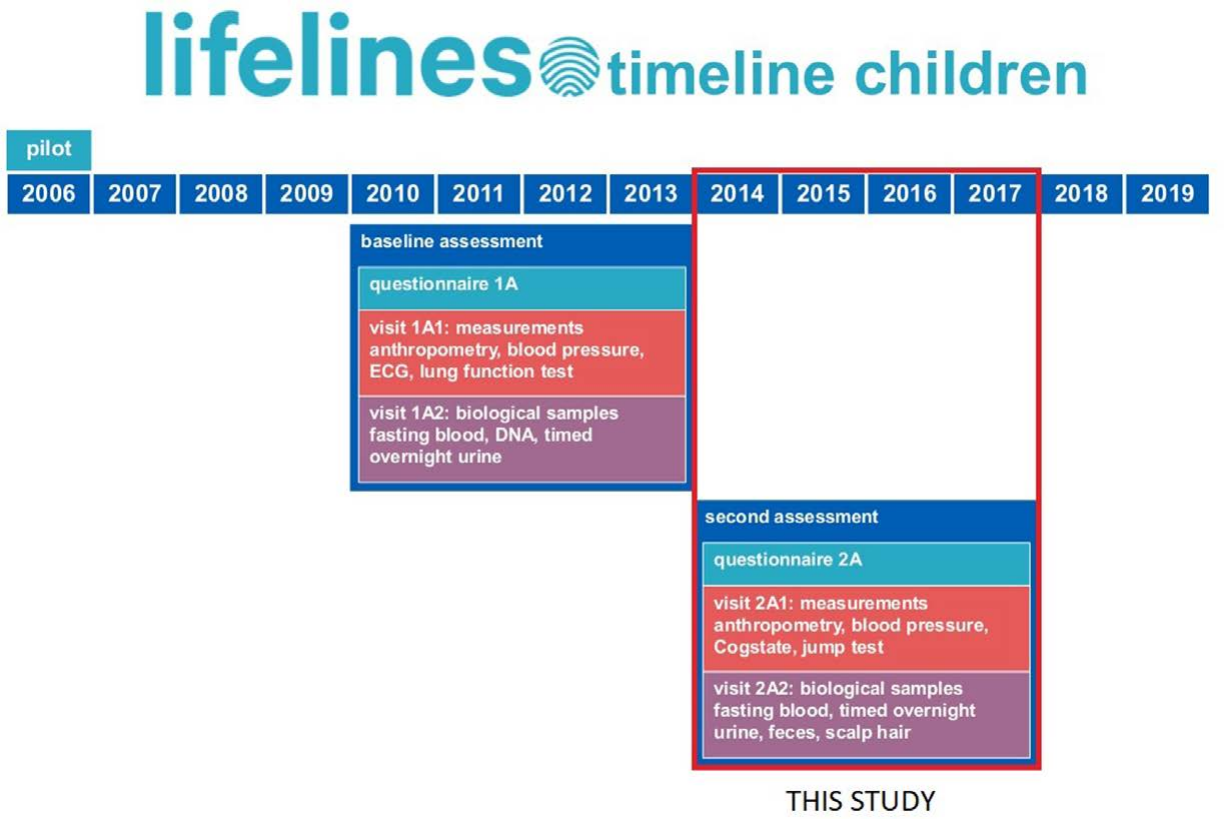
Lifelines timetable.

After assessment “1A” which took place from 2010 to 2014, once every 5 years participants are reinvited to visit a Lifelines location for follow-up assessments and once every 1.5 years they fill out questionnaires on health issues. The second assessment “2A” took place from 2014 to 2018. Within the present study, data from children and adolescents (<18 years) at follow-up assessment “2A” were analyzed based on the fact that during this assessment tinnitus suffering was investigated. For assessment “2A” participants were invited to visit a lifelines location of choice. At this location, multiple measurements were performed (e.g., blood pressure measurements, jump test). Parents/caregivers of young children filled out the questionnaires online before they visited the location. Adolescents (13 to 17 years) filled out the questionnaires on a computer without supervision. Participants were included when complete data on both tinnitus suffering and behavioral assessments were available. Outcomes were included if more than n = 10 participants were available for final analysis (see data assessment for Fig. 1).

### Outcomes

#### Demographic Variables

Data regarding the age, sex, and educational level of the participants were obtained using questionnaires and municipality records. Educational level in adolescents was divided into three groups: High (university, higher vocational education, and pre-university secondary education), medium (senior general secondary education and preparatory secondary vocational education [theoretical pathway]), or low (all other educational subtypes).

#### Prevalence of Tinnitus Suffering

To assess the prevalence of tinnitus suffering in children (4 to 12 years old) parents or caregivers answered the question: *“can you indicate how much your child suffered from this problem?” “Ringing of the ears”* listed as category which needed to be answered. This question is translated from the Dutch sentence *“Wilt u aangeven hoeveel last uw kind het afgelopen jaar van de onderstaande problemen heeft gehad?” “Oorsuizen”.* Adolescents (13 to 17 years old) answered the equivalent question: “Can you indicate how much you suffered from this problem in the past year?” with the subcategory “Ringing ears.” For both children and adolescents, the answer was given on a four-point scale (answer options: not at all, a bit, a lot, or extremely). This question is translated from the Dutch sentence *“Wil je aangeven hoeveel last je het afgelopen jaar van deze problemen hebt gehad?” “Oorsuizen”.*

#### Emotional and Behavioral Outcomes

To assess emotional and behavioral problems, the CBCL (parent/caregiver-reported) and YSR (self-reported) questionnaires were used in respectively children and adolescents. Both questionnaires are validated in English and Dutch (Achenbach et al. 2008; Verhulst & van der Ende 2013). The CBCL includes 113 questions on behavioral problems, and the YSR has 112 questions. Individual questions are rated on a three-point scale (0 = not true, 1 = somewhat/sometimes true, or 2 = very true/often true). Multiple questions are combined to form one of the eight subscales: anxious/depressed (13 questions, e.g., about worries, self-consciousness, fear of going to school), withdrawn/depressed (8 questions e.g., about being sad, withdrawn, or have a lack of energy), somatic complaints (11 questions e.g., about having nightmares, feeling dizzy, eye problems, or vomiting), social- (11 questions e.g., about being lonely, being jealous, getting teased, being clumsy), thought- (15 questions e.g., about harming yourself, strange behavior, or strange ideas), and attention-problems (10 questions e.g., about acting young, failing to finish, lack of concentration, or daydreaming), rule-breaking behavior (17 questions e.g., about drinking alcohol, breaking rules, having bad friends, or stealing), aggressive behavior (18 questions e.g., about: mood changes, getting in fights, being disobedient at school or home). Broadband scales to identify internalizing or externalizing problems are made by combining different subscales. The internalizing behavioral problem scale includes three subscales (anxious/depressed, withdrawn/depressed, somatic complaints), whereas the externalizing behavioral problem scale includes two subscales (rule-breaking behavior, aggressive behavior) (Verhulst & van der Ende 2013; ASEBA n.d.). The raw sum scores are converted to T-scores for which sex-specific rules were used (Achenbach 1999; Verhulst & van der Ende 2013). Sum scores are used within research, whereas T-scores are used to identify problems clinically (Achenbach et al. 2008).

#### Social Functioning Outcome

To assess social functioning in children the parent-reported seven-item PROMIS—peer relationships 7a Parent Proxy SF v1.0 was used (Dewalt et al. 2013). For adolescents, the self-reported eight-item PROMIS—Peer Relationships 8a Pediatric SF v1.0 was used (Dewalt et al. 2013). Both questionnaires are validated in Dutch and are answered on a five-point scale (0 = not at all, 1 = a little bit, 2 = somewhat, 3 = quite a bit, 4 = very much). Raw scores were computed and converted to T-scores (Rothrock et al. 2020). A higher score indicates better social functioning (Haverman et al. 2016; Luijten et al. 2021).

#### Fatigue

To assess fatigue in children the parent-reported 10-item PROMIS fatigue10a Parent Proxy SF v1.0 was used (Lai et al. 2013). For adolescents the self-report 10-item PROMIS-10a fatigue Pediatric SF v1.0 was used (Lai et al. 2013). Both questionnaires are validated in Dutch. Both questionnaires include 10 items with answer options on a five-point scale (0 = not at all, 1 = a little bit, 2 = somewhat, 3 = quite a bit, 4 = very much). Raw scores were computed as sum scores and converted to T-scores; higher scores are indicating more fatigue (Rothrock et al. 2020; Luijten et al. 2021).

#### Concentration Problems and Use of Sleeping Medication

Concentration difficulties were assessed by the following questions “Do(es) your child/you have any of the following learning difficulties?” “Poor concentration or ADHD.” The question needed to be answered by (yes/no/don’t know). Use of sleeping medication was investigated using the statement “I/my child use(s) pills or drops to fall asleep” with answer options”.

#### Data Handling

The data from the Lifelines cohort were analyzed by three authors (S.M.M., J.H.J.D.R., I.S.). Patients were excluded if their age was <4 or >17 years. Another reason for exclusion was either missing of the tinnitus assessment answer or missing of the CBCL/YSR results in the appropriate age range (CBCL 4 to 12 years of age, YSR 13 to 17 years of age).

### Statistical Analysis

For statistical analysis, IBM SPSS statistics version 28.0 was used. Descriptive statistics were performed to summarize demographical outcomes, single-item questionnaires, and Likert-scale questionnaires regarding tinnitus suffering and behavioral outcomes, concentration difficulties, and medication use for children and adolescents separately. Univariable binary logistic regressions were used to assess the association between the sum scores of the domains of the CBCL and YSR, broadband scale outcomes of the CBCL and YSR, T-scores of the PROMIS Questionnaire, and tinnitus impact scores. Tinnitus was dichotomized into: “no tinnitus suffering” and “suffering from tinnitus.” The “suffering from tinnitus” group contained the answer options “suffered a bit,” “ suffered a lot” and “suffered extremely.”

## RESULTS

### Study Participants

**Fig. 2.**
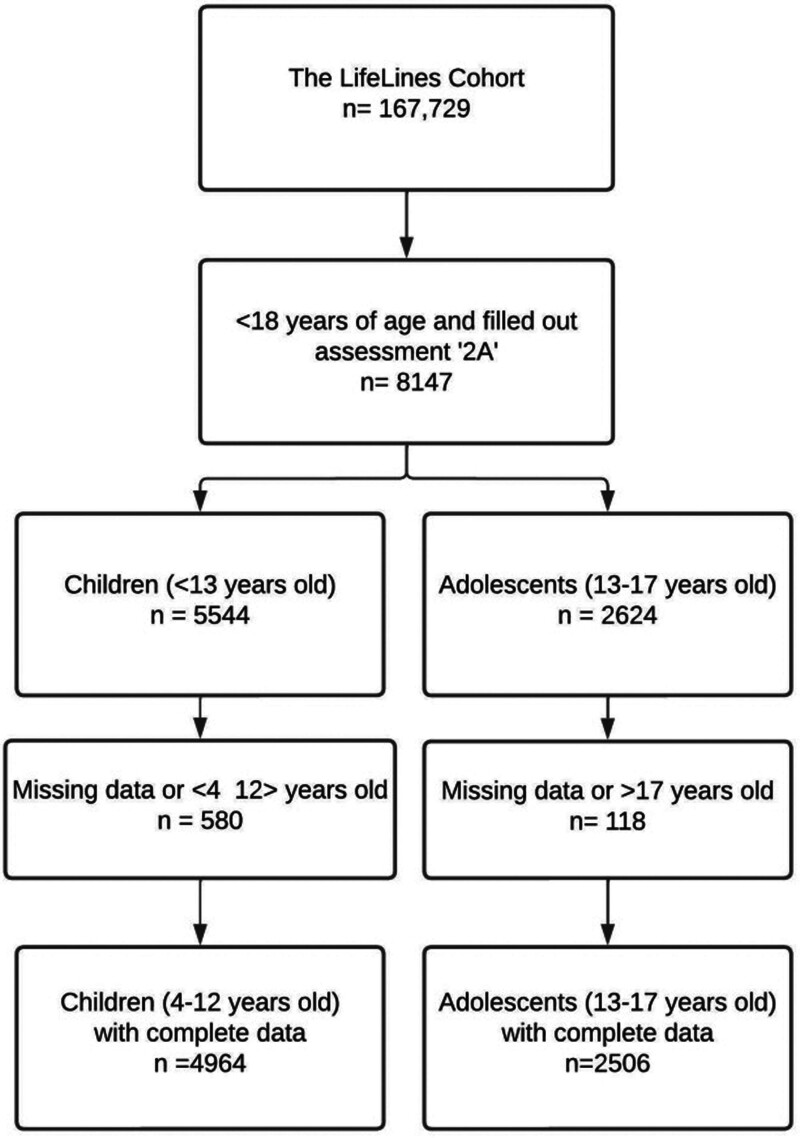
Flowchart.

A total of 4964 children (4 to 12 years old) and 2506 adolescents (12 to 18 years old) were included in the final analysis. A total of 580 (8.5%) children and 118 (4.7%) adolescents were excluded because of their age (<4 years or >18 years) (n = 315 children and n = 44 adolescents) or due to missing data in either one of the questionnaires regarding tinnitus of behavioral outcomes (n = 265 children and n = 74 adolescents). The mean age of the children was 8.0 (SD = 2.8) years and the mean age of the adolescents was 15.0 (SD = 1.3) years. Among the children, 49.8% (n = 2472) were male, while for adolescents, this figure was 47.2% (n = 1183). The educational level of the adolescents was high in 30.0% (n = 752), middle in 51.6% (n = 1293), and low in 18.4% (n = 464) of the participants (Fig. 2). The response rate in the pediatric population for assessment “2A” was 71.8% compared with baseline assessment “1A” (Sijtsma *et al.* 2022). The prevalence of ADHD and sleeping medication use are reported in Table 1.

**TABLE 1.** Baseline characteristics

	Age 4–12		Age 13–17	
	Tinnitus −	Tinnitus +	Tinnitus −	Tinnitus +
N=	4800	164	2187	319
	Mean (SD)		Mean (SD)	
Age	8.0 (2.8)	8.0 (2.8)	15.0 (1.3)	15.1 (1.3)
	N (%)	N (%)	N (%)	N (%)
Sex (male)	2404 (50.1)	69 (42.1)	1039 (47.5)	143 (44.8)
Education				
High	NA	NA	687 (35.3)	86 (30.0)
Middle	NA	NA	927 (47.6)	148 (51.6)
Low	NA	NA	334 (17.1)	53 (18.5)
Tinnitus				
Did not suffer	4800 (96.7)		2187 (87.2)	
Suffered a bit		148 (3.0)		272 (10.9)
Suffered a lot		16 (0.3)		34 (1.4)
Extremely		0.0 (0.0)		13 (0.5)
Concentration problems (ADHD)*				
Yes	414 (8.6)	14 (8.5)	295 (13.5)	59 (18.6)
No	4219 (88.0)	143 (87.2)	1889 (86.5)	258 (81.4)
Sleep medication				
Yes	189 (4.1)	13 (7.9)	92 (4.2)	30 (9.4)
No	4603 (95.9)	150 (92.1)	2094 (95.8)	289 (90.6)
Behavioral Questionnaires	Tinnitus −	Tinnitus +	Tinnitus −	Tinnitus +
N=	4776	163	2182	318
	Mean (SD)	Mean (SD)	Mean (SD)	Mean (SD)
PROMIS fatigue	12.0 (4.5)	13.2 (5.8)	13.94 (6.2)	16.9 (8.1)
PROMIS social	34.5 (5.0)	33.4 (5.1)	32.82 (4.9)	31.9 (5.1)

* Both age groups (n = 168) replied “don’t know”

Age 13–17, education; High, university, higher vocational education and pre-university secondary education; Low, all other educational subtypes; Medium, senior general secondary education and preparatory secondary vocational education (theoretical pathway).

published online ahead of print July 10, 2024

### Tinnitus Suffering and Behavioral Outcomes

In the age group from 4 to 12 years old, 4800 participants (96.7%) answered not to suffer from ringing of the ears, 148 (3.0%) suffered a bit and 16 (0.3%) suffered a lot. None of the participants suffered extremely from ringing in the ears. In the age group from 13 to 17 years old, 2187 (87.2%) of the participants answered not to suffer from ringing of the ears. Among them, 272 (10.9%) of the participants suffered a bit, 34 (1.4%) of the participants suffered a lot and 13 (0.5%) of the participants suffered extremely. Mean scores, the SD, and range for the behavioral outcomes of the PROMIS questionnaire for both age groups are reported in Table 1. Mean scores, SD, and range for both raw scores and T-scores for the behavioral outcomes using the CBCL and YSR questionnaires are reported in Table 2.

**TABLE 2.** Baseline continuation

Variable							
CBCL Syndrome Scales	Patients (n)	Raw Score (Mean [SD])	Range (Min-Max)	Min-Max Score	T Score (Mean [SD])	Range (Min-Max)	Min-Max Score
I Anxious/depressed	4964	1.4 (2.0)	0–18	0–26	51.5 (3.5)	50–84	50–100
II Withdrawn/depressed	4964	0.8 (1.4)	0–14	0–16	53.5 (5.7)	50–94	50–100
III Somatic complaints	4964	1.4 (1.7)	0–13	0–22	54.1 (5.6)	50–82	50–100
IV Social problems	4964	1.2 (1.7)	0–16	0–22	51.7 (3.3)	50–85	50–100
V Thought problems	4964	1.2 (1.7)	0–13	0–30	52.5 (4.5)	50–77	50–100
VI Attention problems	4964	2.3 (2.5)	0–18	0–20	52.5 (3.9)	50–93	50–100
VII Rule-breaking behavior	4964	0.5 (1.0)	0–10	0–34	51.1 (2.4)	50–74	50–100
VIII Aggressive behavior	4964	2.6 (3.3)	0–25	0–36	52.0 (4.0)	50–83	50–100
IX Other problems	4964	2.2 (2.0)	0–13	0–34			
Internalizing problems	4964	3.6 (4.0)	0–35	0–64	45.7 (9.1)	33–78	33–100
Externalizing problems	4964	3.1 (3.9)	0–31	0–70	44.1 (8.9)	33–75	33–100
Total score	4964	13.6 (12.1)	0–101	0–240	43.7 (9.6)	24–75	24–100
YSR Syndrome Scales	Patients (n)	Raw Score (Mean [SD])	Range (Min-Max)	Min-Max Score	T Score (Mean [SD])	Range (Min-Max)	Min-Max Score
I Anxious/depressed	2506	2.3 (2.7)	0–18	0–26	51.6 (3.6)	50–79	50–100
II Withdrawn/depressed	2506	3.2 (1.9)	1–12	0–16	54.5 (5.0)	50–83	50–100
III Somatic complaints	2506	2.3 (2.4)	0–16	0–20	53.2 (4.8)	50–86	50–100
IV Social problems	2506	1.4 (1.7)	0–11	0–22	51.3 (2.8)	50–73	50–100
V Thought problems	2506	5.7 (2.6)	0–20	0–24	58.2 (5.5)	50–90	50–100
VI Attention problems	2506	4.0 (2.7)	0–13	0–18	52.9 (4.3)	50–78	50–100
VII Rule-breaking behavior	2506	6.8 (2.3)	0–17	0–30	59.7 (4.6)	50–77	50–100
VIII Aggressive behavior	2506	6.6 (2.7)	0–17	0–34	53.7 (4.1)	50–75	50–100
XI Other problems	2506	2.5 (2.0)	0–12	0–20			
Internalizing problems	2506	7.8 (5.5)	1–36	0–62	47.6 (5.2)	32–75	33–100
Externalizing problems	2506	13.3 (4.4)	0–32	0–64	56.0 (5.2)	29–74	33–100
Total score	2506	33.3 (14.1)	1–97	0–210	49.7 (6.6)	29–74	24–100

### Associations Between Behavioral Outcomes and Tinnitus Suffering

#### CBCL Outcomes (Age 4 to 12 Years Old)

Univariate binary logistic regressions were used to analyze the association between tinnitus suffering and scores of the domains of CBCL. They were analyzed per sex. Suffering from tinnitus was associated with the subscales “Anxious/depressed”, “Withdrawn/depressed”, “Somatic complaints” and “Social problems” for both boys and girls from 4 to 12 years old using the CBCL. The subscales “Thought problems” and “Attention problems” were associated with tinnitus suffering in girls only. “Internalizing problems” and the total CBCL sum score were associated with tinnitus suffering for both of the sexes (Table 3).

**TABLE 3.** Odds ratio of tinnitus suffering and behavioral outcomes raw scores (age group 4–12 yrs old)

Variable	Sex (M/F)	N (No Tinnitus/Yes Tinnitus)	Tinnitus: Yes (Mean [SD])	Tinnitus: No (Mean [SD])	OR (95% CI, *p*)
CBCL syndrome scales					
I Anxious/depressed	M	2473 (2404/69)	2.0 (2.5)	1.3 (1.9)	1.14 (1.05–1.25, 0.01)
I Anxious/depressed	F	2491 (2396/95)	2.4 (2.6)	1.5 (2.0)	1.17 (1.08–1.26, <0.01)
II Withdrawn/depressed	M	2473 (2404/69)	1.3 (1.7)	0.9 (1.5)	1.14 (1.01–1.30, 0.03)
II Withdrawn/depressed	F	2491 (2396/95)	1.0 (1.4)	0.7 (1.2)	1.18 (1.03–1.35, 0.02)
III Somatic complaints	M	2473 (2404/69)	1.7 (2.2)	1.2 (1.6)	1.17 (1.04–1.32, 0.01)
III Somatic complaints	F	2491 (2396/95)	2.8 (2.6)	1.5 (1.8)	1.28 (1.19–1.39, <0.01)
IV Social problems	M	2473 (2404/69)	1.8 (2.2)	1.2 (1.7)	1.17 (1.06–1.29, <0.01)
IV Social problems	F	2491 (2396/95)	1.5 (1.9)	1.1 (1.8)	1.11 (1.01–1.22, 0.04)
V Thought problems	M	2473 (2404/69)	1.5 (2.0)	1.2 (1.7)	1.10 (0.97–1.23, 0.13)
V Thought problems	F	2491 (2396/95)	1.9 (2.1)	1.1 (1.6)	1.23 (1.13–1.35, <0.01)
VI Attention problems	M	2473 (2404/69)	3.0 (2.6)	2.6 (2.6)	1.06 (0.97–1.15, 0.20)
VI Attention problems	F	2491 (2396/95)	2.5 (2.6)	2.0 (2.3)	1.09 (1.01–1.18, 0.03)
VII Rule-breaking behavior	M	2473 (2404/69)	0.8 (1.2)	0.6 (1.1)	1.16 (0.96–1.40, 0.12)
VII Rule-breaking behavior	F	2491 (2396/95)	0.6 (0.9)	0.4 (0.8)	1.16 (0.95–1.43, 0.14)
VIII Aggressive behavior	M	2473 (2404/69)	3.3 (4.0)	2.8 (3.5)	1.03 (0.97–1.10, 0.32)
VIII Aggressive behavior	F	2491 (2396/95)	2.7 (3.0)	2.3 (3.0)	1.04 (0.98–1.11, 0.17)
IX Other problems	M	2473 (2404/69)	2.8 (1.9)	2.4 (2.1)	1.09 (0.98–1.21, 0.11)
IX Other problems	F	2491 (2396/95)	2.3 (2.1)	2.0 (2.0)	1.07 (0.97–1.18, 0.15)
Externalizing problems	M	2473 (2404/69)	4.1 (4.9)	3.5 (4.3)	1.03 (0.98–1.08, 0.23)
Externalizing problems	F	2491 (2396/95)	3.3 (3.5)	2.8 (3.5)	1.04 (0.99–1.09, 0.13)
Internalizing problems	M	2473 (2404/69)	5.0 (5.4)	3.3 (3.9)	1.08 (1.03–1.12, <0.01)
Internalizing problems	F	2491 (2396/95)	6.2 (5.2)	3.7 (4.0)	1.11 (1.07–1.15, <0.01)
Sum score	M	2473 (2404/69)	18.2 (14.5)	14.1 (12.5)	1.02 (1.00–1.04, 0.01)
Sum score	F	2491 (2396/95)	17.7 (12.6)	12.7 (11.6)	1.03 (1.01–1.04, <0.01)

### YSR (Age 13 to 17 Years Old)

Suffering from tinnitus was associated with all subscales of the YSR questionnaire for both sexes in the age group 13 to 17 years. Tinnitus suffering was associated with internalizing and externalizing problems for both sexes. “Anxious/depressed”, “Withdrawn/depressed”, “Somatic complaints”, “Social problems”, “Thought problems”, “Attention problems”, “Rule breaking behavior”, “Aggressive behavior”, “Other problems” and the total YSR sum score were also all associated (Table 4).

**TABLE 4.** Odds ratio of tinnitus suffering and behavioral outcomes raw scores (age group 13–17 yrs old)

Variable	Sex (M/F)	N (No Tinnitus—Yes Tinnitus)	Tinnitus: Yes (Mean [SD])	Tinnitus: No (Mean [SD])	OR (95% CI, *p*)
YSR syndrome scales					
I Anxious/depressed	M	1182 (1039/143)	2.3 (2.3)	1.6 (2.0)	1.15 (1.07–1.23 <0.01)
I Anxious/depressed	F	1324 (1148/176)	4.0 (3.5)	2.7 (2.8)	1.14 (1.09–1.19 <0.01)
II Withdrawn/depressed	M	1182 (1039/143)	3.9 (1.9)	3.2 (1.9)	1.18 (1.09–1.23 <0.01)
II Withdrawn/depressed	F	1324 (1148/176)	3.8 (2.1)	3.0 (1.8)	1.24 (1.15–1.34 <0.01)
III Somatic complaints	M	1182 (1039/143)	2.3 (2.2)	1.5 (1.9)	1.21 (1.12–1.31 <0.01)
III Somatic complaints	F	1324 (1148/176)	4.0 (3.1)	2.7 (2.6)	1.18 (1.12–1.24 <0.01)
IV Social problems	M	1182 (1039/143)	1.8 (1.8)	1.3 (1.5)	1.19 (1.08–1.32 <0.01)
IV Social problems	F	1324 (1148/176)	2.2 (2.1)	1.4 (1.6)	1.26 (1.16–1.36 <0.01)
V Thought problems	M	1182 (1039/143)	6.5 (2.4)	5.0 (2.3)	1.25 (1.17–1.34 <0.01)
V Thought problems	F	1324 (1148/176)	7.4 (2.9)	5.9 (2.6)	1.21 (1.15–1.28 <0.01)
VI Attention problems	M	1182 (1039/143)	4.7 (2.7)	3.7 (2.6)	1.15 (1.08–1.23 <0.01)
VI Attention problems	F	1324 (1148/176)	5.5 (2.8)	4.1 (2.7)	1.21 (1.14–1.28 <0.01)
VII Rule-breaking behavior	M	1182 (1039/143)	7.7 (2.8)	6.5 (2.4)	1.19 (1.11–1.28 <0.01)
VII Rule-breaking behavior	F	1324 (1148/176)	7.5 (2.3)	6.8 (2.2)	1.16 (1.08–1.24 <0.01)
VIII Aggressive behavior	M	1182 (1039/143)	7.7 (2.9)	6.6 (2.8)	1.14 (1.08–1.21 <0.01)
VIII Aggressive behavior	F	1324 (1148/176)	7.4 (3.1)	6.3 (2.2)	1.15 (1.08–1.21 <0.01)
IX Other problems	M	1182 (1039/143)	3.1 (1.8)	2.4 (1.8)	1.21 (1.11–1.32 <0.01)
IX Other problems	F	1324 (1148/176)	3.4 (2.3)	2.5 (2.0)	1.21 (1.13–1.30 <0.01)
Internalizing problems	M	1182 (1039/143)	8.5 (5.6)	6.2 (4.5)	1.09 (1.06–1.13 <0.01)
Internalizing problems	F	1324 (1148/176)	11.9 (6.8)	8.5 (5.6)	1.09 (1.06–1.11 <0.01)
Externalizing problems	M	1182 (1039/143)	15.4 (5.1)	13.1 (4.6)	1.10 (1.06–1.14 <0.01)
Externalizing problems	F	1324 (1148/176)	14.9 (4.7)	13.1 (4.1)	1.10 (1.06–1.14 <0.01)
Sum score	M	1182 (1039/143)	38.1 (13.8)	30.3 (12.9)	1.04 (1.03–1.05 <0.01)
Sum score	F	1324 (1148/176)	43.0 (16.6)	33.9 (13.9)	1.04 (1.03–1.05 <0.01)

### Associations Between Fatigue, Social Functioning, and Tinnitus Suffering

Suffering from tinnitus was associated with higher scores for fatigue for both children and adolescents using the PROMIS questionnaire (age = 4 to 12: odds ratio [OR] = 1.03 [1.01 to 1.05, *p* < 0.01, n = 4939], age 13 to 17: OR = 1.04 [1.03 to 1.05, *p* < 0.01, n = 2500]). Suffering from tinnitus was also associated with lower scores for social functioning using the PROMIS questionnaire (age 4 to 12: OR = 0.98 [0.96–0.99, *p* ≤ 0.01, n = 4941], age 13 to 17: OR = 0.98 [0.96–0.99, *p* ≤ 0.01, n = 2497]).

## DISCUSSION

In this cross-sectional study, we investigated the prevalence of tinnitus suffering and behavioral outcomes in a cohort of children and adolescents in a nonclinical setting. The prevalence of children who report to suffer from tinnitus was 3.3 and 12.8% in children and adolescents, respectively. 0.3% of the children and 1.9% of the adolescents reported to suffer a lot or extremely. For children, internalizing problems (anxiety, depression, somatic complaints) were associated with tinnitus suffering. In adolescents, both internalizing and externalizing problems (rule-breaking behavior and aggressive behavior) were associated with tinnitus suffering.

In a systematic review from 2016 tinnitus prevalence ranged between 4.7 and 46% in the general pediatric population without hearing loss (age range 4 to 19 years) (Rosing et al. 2016). More recent studies report different results depending on the age of the participants and the recruitment location. Two studies investigated the age group 12 to 18 years old and found a tinnitus prevalence of 18.0 (Kim 2018) and 32.3% (Kim et al. 2017). Severe tinnitus was present in 1.3 (Kim et al. 2017) and 18.1% (Kim 2018) of those with tinnitus. Humphriss et al. (2016) investigated 7092 11-year-old children and found a tinnitus prevalence of 28.1% and “severe tinnitus” in 3.1% of the children. In one large study that investigated tinnitus in school children, a tinnitus prevalence of 6.0% was found in a sample of 15.199 primary school children who were 7- and 12-year of age (Raj-Koziak et al. 2021). Other studies that investigated school children with varying age ranges found a prevalence of 46 and 66.9% (Nemholt et al. 2020; Rhee et al. 2020). (See supplementary file: overview of published studies since Rosing *et al.* in Supplemental Digital Content, http://links.lww.com/EANDH/B434, including the used definition of tinnitus and study populations.)

The use of different index questions to identify those with tinnitus (Rosing et al. 2016; Nemholt et al. 2020; Raj-Koziak et al. 2021), different cutoff values to define tinnitus or differences in how the level of suffering was scored will strongly influence the outcome; a problem that is also known in the adult population (Rademaker et al. 2021). The assessment of tinnitus in very young children is frequently done by proxy, which means caregivers are asked questions about tinnitus experienced by their children. However, it is known that parents/caregivers tend to be unaware of the tinnitus of their child, which causes them to underreport this symptom (Raj-Koziak et al. 2021). In other fields, poor agreement between scores of children self-report and parent by proxy report have also been noticed (Eiser & Morse 2001; Vetter et al. 2012). In this study, parents filled out the questionnaires if their child was younger than 13 years of age, whereas the age group from 13 to 17 filled out the tinnitus questionnaire themselves. This could explain the remarkable difference in tinnitus prevalence when 12 and 13-year-olds are compared (12-year-olds: 3, 4% 13-year-olds: 11, 3%). Also, the onset of puberty could be of influence.

Despite the differences in study design of pediatric tinnitus studies, all studies report that the prevalence of tinnitus rises with age, similar to the findings of our study (Kim 2018; Nemholt et al. 2020; Rhee et al. 2020). Besides a better understanding of the asked questions to study tinnitus by older children, other explanations for this observation do exist. Noise-induced hearing loss, a risk factor for tinnitus presence in adults, is more present in adolescents in comparison to children due to headphone use and more exposure to loud environments like discotheques and concerts (Bohlin & Erlandsson 2007; Båsjö et al. 2016; Kim 2018; Choi et al. 2021). Another explanation is that young children consider tinnitus as a normal event and only experience this as abnormal or as a burden from a later age. This could be related to the idea that young children are more easily distracted by external stimuli and therefore are not bothered by the symptom (Viani 1989). It could be worthwhile to study the pathway of being bothered by the experience of tinnitus in future studies.

The association between emotional factors and tinnitus should also be taken into account. In adult tinnitus patients psychiatric symptoms like depressive feelings or anxiety have frequently been reported (Trevis et al. 2018; Meijers et al. 2022). In pediatric studies the evidence is limited. Two population-based studies found a significant correlation between tinnitus and depressive symptoms or a depression in either 15 to 16-year-olds (Brunnberg et al. 2008) and 12 to 18-year-olds (Kim 2018). The study of Rhee et al. (2020) reported depressive feelings in 19.7% of the participants when investigating tinnitus in the age groups of 12 to 13 and 15 to 16 years old and Kim et al. (2017) found a significant association between tinnitus presence and depression using the Children Depression Inventory in children between 10 and 12 years of age, both at schools (Kovacs 1992). Anxiety is found to be significantly associated with the presence of tinnitus in four studies that investigated this in 10 to 12-year-old children (Kim et al. 2012), 15 to 16-year-old children (Brunnberg et al. 2008), 9 to 16-year-old children (Holgers & Juul 2006), and 12 to 17-year-old children (Kim et al. 2015). In this study, symptoms of anxiety and depression were measured using different syndrome scales of either the CBCL or YSR. Both were associated with tinnitus suffering in children and adolescents, although the effect sizes were small. Earlier research proposed that trait anxiety (e.g., the fairly stable personality characteristic of responding with anxiety to stimuli) (Taylor & Deane 2002), but not state anxiety (e.g., a transient/temporary condition or emotional state characterized by subjective, consciously perceived feelings of tension/anxiety), is associated with presence and frequency of tinnitus, and may result in greater distress (Kim et al. 2012).

Tinnitus suffering was also associated with somatic complaints in children and adolescents in this study. Only the study of Rhee et al. (2020) and the study of Brunnberg et al. (2008) previously reported on this topic in adolescents and found associations between tinnitus and somatic complaints like stomach troubles, abdominal pain, feeling bloated and reflux (Brunnberg et al. 2008; Rhee et al. 2020). Somatization and even somatoform disorders are also associated with tinnitus in adults but cause-effect direction remains unclear (Sahin et al. 2016; Meijers et al. 2020).

Attention problems were associated with tinnitus suffering in girls and adolescents of both sexes using CBCL or YSR in this study. The self-reported or parent-reported prevalence of AHDH was 8.5% for children who suffered from tinnitus and 18.6% for adolescents who suffered from tinnitus. This is more than double the prevalence which is reported by the Dutch Bureau of Statistics (Netherlands n.d.). The design of this question could explain these differences. In the questionnaire adults/caregivers or adolescents were asked if their child or if they themselves suffered from “concentration problems (ADHD).” Also, participants with a minor concentration problems could have falsely answered the question with a “yes.” The only other study that investigated ADHD or attention problems in children with tinnitus also found a significant association between tinnitus in female adolescents (Kim et al. 2017). Studies that investigated pediatric tinnitus patients who visited an outpatient clinic for their tinnitus found that 12 to 32.1% had concentration problems (Szibor et al. 2017; Chan et al. 2018).

In this study, associations between tinnitus suffering and internalizing and externalizing problems were found in adolescents, whereas in children only internalizing problems are associated with tinnitus. No other pediatric tinnitus study has investigated this topic so it is not possible to compare these results. These differences between children and adolescents might be due to the physical and emotional changes that adolescents undergo that might interfere with emotion regulation and tinnitus perception (Ahmed et al. 2015; Kim et al. 2017).

Tinnitus suffering was found to be associated with fatigue in this study. Sleep medication use was also higher in tinnitus sufferers compared with non-tinnitus sufferers. Tiredness or sleep disturbance has been mentioned in multiple studies that investigate pediatric tinnitus. Brunnberg et al. (2008) found a significant correlation between tinnitus distress and tiredness in 15 to 16-year-old adolescents. Kim et al. (2012) reported significantly more sleep disturbance in 10 to 12-year-old children with tinnitus. Sleeping problems were also reported in 28.6% of the children between 3.6 and 17.9 years of age who visited an ENT outpatient clinic with the main complaint of tinnitus (Chan et al. 2018).

There are some limitations to this study. The Lifelines cohort study does not perform a hearing test in pediatric participants, so hearing loss, a known risk factor for tinnitus could not be investigated. Also, the question regarding tinnitus does focus on the experienced tinnitus suffering, not on the presence or duration of tinnitus. These additional questions could provide extra information and help to better distinguish between the prevalence of tinnitus presence and tinnitus suffering in children and adults. The cross-sectional design of this study hinders to establish a cause-effect relationship between the found outcomes or to differentiation between state and trait anxiety for now. However, the Lifelines cohort study is designed to follow the participants for 30 years, so future studies might be able to shine more light on this matter. The outcome of our study, tinnitus suffering, was originally categorized into four categories (did not suffer, suffered a bit, suffered a lot, suffered extremely), we choose to dichotomize the outcome, and use logistic regression because the number of children in each category was low. While we could not have done the analyses with such low numbers in each category, we now assumed that the children could be categorized as suffering, which might have been an oversimplification. Also, there is the possibility that even if a person experiences tinnitus, they may not suffer from it.

Due to the lack of an international consensus regarding the definition of pediatric tinnitus and ways to identify pediatric tinnitus, results between different studies are hard to compare. Research groups should focus on reaching consensus on this topic and the development of a pediatric tinnitus questionnaire. This could result in better comparability between studies and more useful results. The rise in prevalence of tinnitus with age could possibly be explained by the changes in emotional regulation in adolescents. More insight into this phenomenon could provide essential clues for researchers to answer the question of why tinnitus in some patients devastates their lives while others are not bothered by it.

## CONCLUSION

In this study, we investigated emotional and behavioral outcomes using validated questionnaires in nonclinical children with tinnitus. The prevalence of tinnitus and the impact of tinnitus on daily life rises with age. Tinnitus is associated with internalizing and externalizing problems in adolescents and with internalizing problems in children. Future research should focus on achieving a consensus for the definition of pediatric tinnitus and the development of a pediatric tinnitus questionnaire. The findings of this study could aid this.

## ACKNOWLEDGMENTS

The Lifelines initiative has been made possible by subsidy from the Dutch Ministry of Health, Welfare and Sport, the Dutch Ministry of Economic Affairs, the University Medical Center Groningen (UMCG), Groningen University, and the Provinces in the North of the Netherlands (Drenthe, Friesland, Groningen). The Lifelines Cohort Study is approved by the Medical Ethical Committee of the University Medical Center Groningen, The Netherlands under number 2007/152 and is performed according to the principles of the Declaration of Helsinki.

## Supplementary Material


