# Supplementary material for: The Lifelines Cohort Study: Prevalence of Tinnitus Associated Suffering and Behavioral Outcomes in Children and Adolescents
Source: Ear Hear. 2024 Jul 10;45(6):1517–26. doi: 10.1097/AUD.0000000000001538 (PMC11487026; doi:10.1097/AUD.0000000000001538)
Supplement: Supplementary file 1 [file aud-45-1517-s001.pdf]

**Supplementary file: Studies that report pediatric tinnitus prevalence since Rosing *et al.* 2016 in English literature.**						
**Study, year**	**design**	**Age (years)**	**N (tin)**	**Prevalence tinnitus (%)**	**Prevalence 'severe tinnitus' (%)**	**location**
Humphriss, 2016 (5)	PPBS	11	7092 (1992)	28.1	3.1	Population
Kim, 2016 (31)	CSC	12-17	962 (311)	32.3	1.3 (THI 58-100)	School
Adegbniji, 2018 (18)	PCS	<18	2123 (132)	6.2**	NR	Outpatient
Kim, 2018 (6)	PPBS	12-18	1587	18.0	18.1 (of the included tinnitus patients)	Population
Lee, 2018 (44)	PPBS	12-18	2782 (486)	17.5	3.3	Population
Rhee, 2020 (8)	PCS	12-13<br>15-16*	1593	46	9.1	School
Nemholt, 2020 (10)	CSC	10.9-16.6	501	66.9	34.6% (of the included tinnitus patients)	
Swierniak, 2020 (45)	NR	11-12 PMP+<br>11-12 PMP-	849 (326)<br>189 (50)	38.4<br>24.0	NR<br>NR	School
Raj-Koziak, 2020 (7)	PPBS	11-13	43064	3.1		School
This study, 2023	PPBS	4-12<br>13-17	4964(164)<br>2506 (319)	3.3<br>12.8	0.3<br>1.9	Population

SR: systematic review, RCS: retrospective cohort study, PPBS: population based prospective study, PCS: prospective cohort study Cross-sectional cohort study *= no seperate information for both age groups **= of the patients who visited an ENT outpatient clinic. PMP+ personal music player NR = not reported. Y= yes, N=No THI= Tinnitus Handicap Inventory (Newman *et al.* 1996)

**Supplementary file continuation: tinnitus definitions**
**Definition tinnitus:**<br>Humphriss *et al*.: ' Do you ever get noises in your ears? (not associated with noise exposure)'. (Y/N)<br>Kim 2016 *et al*.: NR<br>Abegbniji *et al*.: NR (patients were referred to the ENT clinic with complaints of tinnitus)<br>Kim 2018 *et al*.: 'In the past 12 months, have you been bothered by buzzing in your ears?' (Y/N)<br>Lee 2018 *et al*.: 'Have you heard any ringing, buzzing, roaring, or hissing sounds without an external acoustic source in the past year? (Y/N)<br>Rhee 2020 *et al*.: 'The presence or absence of tinnitus' (no answer option reported)<br>Nemholt *et al*.: Q1: 'After listening to loud music or other loud sounds or noise, have you heard any sort of sounds in your head or ear even after that the loud music or noise has been turned off?' (Y/N) (Q2) 'Do you ever get noise in your head or ears without first having listened to loud music or other sounds?' (Y/N)<br>Swierniak *et al*.: 'Do you hear tinnitus, whistles, squeaks or other sounds when in quiet?' (Y/N)<br>Raj-Koziak *et al*.: parents: 'Does your child complain of tinnitus in their ears/head when in quiet' (very often, often, rarely, or never)<br>Raj-Koziak *et al*.: Children: Do you hear tinnitus, whistles, or squeaks as you are falling asleep or when it is quiet in your room? (yes, all the time; yes, periodically and for more than 5 minutes; yes, but only for a very short time; no)<br>This study: 'Can you indicate how much your child suffered from the types of physical pain listed below in the past year?' ('ringing ears') (not at all, a bit, a lot or extremely)
Definition severe tinnitus:<br>Humphriss *et al*.: Do the noises bother you? (Not bothered, slightly bothered, severely bothered)<br>Kim 2016 *et al*.: using the Tinnitus Handicap Inventory<br>Abegbniji *et al*.: NR<br>Kim 2018 *et al*.: How much of a problem is this ringing in your ears?" (no problem, bothering, having trouble sleeping)<br>Lee 2018 *et al*.: "Do these sounds bother you?" (No, A little annoying, Very annoying and disrupting sleep)<br>Rhee 2020 *et al*.: No problem, I am annoyed and bothered, and I find it hard to sleep.<br>Nemholt *et al*.: (Q4) Do the noises bother you? (Y/N)<br>Raj-Koziak *et al*.: If the parents answered very often or often, and the children answered yes, all the time; yes, periodically and for over than 5 minutes they were considered positive outcomes, i.e. indicating the presence of tinnitus.<br>This study: 'a lot or extremely' indicated severe tinnitus.
